# AtWRKY22 promotes susceptibility to aphids and modulates salicylic acid and jasmonic acid signalling

**DOI:** 10.1093/jxb/erw159

**Published:** 2016-04-23

**Authors:** Karen J. Kloth, Gerrie L. Wiegers, Jacqueline Busscher-Lange, Jan C. van Haarst, Willem Kruijer, Harro J. Bouwmeester, Marcel Dicke, Maarten A. Jongsma

**Affiliations:** ^1^Laboratory of Entomology, Wageningen University and Research Centre, PO Box 16, 6700 AA Wageningen, The Netherlands; ^2^Laboratory of Plant Physiology, Wageningen University and Research Centre, PO Box 16, 6700 AA Wageningen, The Netherlands; ^3^Plant Research International, Business Unit Bioscience, Wageningen University and Research Centre, PO Box 16, 6700 AA Wageningen, The Netherlands; ^4^Plant Research International, Business Unit Biointeractions & Plant Health, Wageningen University and Research Centre, PO Box 16, 6700 AA Wageningen, The Netherlands; ^5^Biometris, Wageningen University and Research Centre, PO Box 16, 6700 AA Wageningen, The Netherlands

**Keywords:** *Arabidopsis thaliana*, mechanostimulation, *Myzus persicae*, plant–insect interaction, plant resistance to aphids, touch, transcription factors.

## Abstract

Aphids induce many transcriptional perturbations in their host plants, but the signalling cascades responsible and the effects on plant resistance are largely unknown. Through a genome-wide association (GWA) mapping study in *Arabidopsis thaliana*, we identified *WRKY22* as a candidate gene associated with feeding behaviour of the green peach aphid, *Myzus persicae*. The transcription factor WRKY22 is known to be involved in pathogen-triggered immunity, and *WRKY22* gene expression has been shown to be induced by aphids. Assessment of aphid population development and feeding behaviour on knockout mutants and overexpression lines showed that WRKY22 increases susceptibility to *M. persicae* via a mesophyll-located mechanism. mRNA sequencing analysis of aphid-infested *wrky22* knockout plants revealed the up-regulation of genes involved in salicylic acid (SA) signalling and down-regulation of genes involved in plant growth and cell-wall loosening. In addition, mechanostimulation of knockout plants by clip cages up-regulated jasmonic acid (JA)-responsive genes, resulting in substantial negative JA–SA crosstalk. Based on this and previous studies, WRKY22 is considered to modulate the interplay between the SA and JA pathways in response to a wide range of biotic and abiotic stimuli. Its induction by aphids and its role in suppressing SA and JA signalling make *WRKY22* a potential target for aphids to manipulate host plant defences.

## Highlight

The transcription factor WRKY22 increases susceptibility to aphids in Arabidopsis via the suppression of salicylic acid signalling.

## Introduction

As plants are sessile organisms in often dynamically changing environments, plasticity is fundamental to survival. Transcriptional regulation plays an important role in how plants cope with environmental stimuli. In Arabidopsis approximately 50 transcription factor families have been identified, accounting for approximately 2000 genes (Guo *et al.*, 2005; Mitsuda and Ohme-Takagi, 2009). Together with signal perception and transduction elements, these transcription factors participate in complex and dynamic networks that regulate developmental processes and responses to (a)biotic stress. Insect infestations are typical situations that require quick transcriptional reprogramming in order to mount an effective defence response. Aphids are phloem-feeding insects that manoeuvre their piercing–sucking mouthparts between cells and reach the vascular bundle without inflicting major physical damage (Minks and Harrewijn, 1989). They are vectors of many plant viruses and deprive the plant of photoassimilates. Aphids cause strong transcriptional perturbations in plants, inducing or repressing up to several thousand genes, whereas other insects such as caterpillars and cell-content feeders alter the expression of only up to several hundred genes (De Vos *et al.*, 2005; Kusnierczyk *et al.*, 2007; Barah *et al.*, 2013; Dubey *et al.*, 2013; Kerchev *et al.*, 2013; Appel *et al.*, 2014; Foyer *et al.*, 2015). An open question is, however, whether these transcriptional changes lead to enhanced resistance to aphids or whether they are unsuccessful or even counter-effective modulations. Aphids are known to secrete effectors via their saliva into the apoplast and the vascular bundle (Rodriguez and Bos, 2012), and might be able to manipulate the host plant physiology for their own benefit. In this study, genome-wide association (GWA) mapping revealed *WRKY22* (*At4g01250*) as one of the candidate genes for affecting feeding behaviour of the generalist aphid *Myzus persicae* (Sulzer) on *Arabidopsis thaliana*. WRKY22 is a member of the WRKY transcription factor family, which was discovered in the 1990s and named after its binding affinity to the W-box promoter motif (Eulgem *et al.*, 2000). WRKY22 and its homologue WRKY29 are part of group IIe WRKYs and are both established markers of pathogen-triggered immunity (PTI). Pathogen-associated molecular patterns (PAMPs) such as flagellin, chitin and cellulysin are recognized elicitors of the mitogen-activated protein kinase (MAPK) cascade that induce *WRKY22* and *WRKY29* within 30min post-inoculation (Asai *et al.*, 2002; Dong *et al.*, 2003; Navarro *et al.*, 2004; Mészáros *et al.*, 2006; Thilmony *et al.*, 2006; Schikora *et al.*, 2011; González-Lamothe *et al.*, 2012; Shi *et al.*, 2015). In general, PTI results in the accumulation of reactive oxygen species and callose deposition and involves salicylic acid (SA), jasmonic acid (JA) and ethylene (ET) signalling (Yi *et al.*, 2014). Although the exact role of WRKY22 and WRKY29 in PTI is unknown, WRKY22 has been shown to be required for resistance to the hemibiotrophic pathogen *Pseudomonas syringae* (Hsu *et al.*, 2013) and WRKY29 has been described as conferring resistance to *P. syringae* as well as to the necrotrophic pathogen *Botrytis cinerea* (Asai *et al.*, 2002). In this study, we assessed the involvement of WRKY22 in plant resistance to *M. persicae* aphids and its downstream transcriptional effects.

## Materials and methods

### Plants and insects

A collection of 344 natural accessions of *A. thaliana* was obtained from the ABRC Stock Center (Baxter *et al.*, 2010). This set was selected in a previous study to represent most intraspecific genetic variation and minimal redundancy (Platt *et al.*, 2010), and was genotyped for 214000 single nucleotide polymorphism (SNPs) with AtSNPtile1 arrays (Atwell *et al.*, 2010; Li *et al.*, 2010; Horton *et al.*, 2012). Transfer (T)-DNA lines SALK_094892 (*wrky22-3*) and SALK_098205 (*wrky22-4*), and TRANSPLANTA-inducible overexpression lines TPT_4.01250.1C and TPT_4.01250.1E were obtained from NASC (Coego *et al.*, 2014). Seeds were cold stratified for 72h at 4 °C before they were sown in pots (5cm diameter) with pasteurized (4h at 80 °C) Arabidopsis potting soil (Lentse Potgrond, Lent, The Netherlands) in a climate room at 24±1 °C, 50–70% relative humidity, 8 h–16h light–dark photoperiod, and a light intensity of 200 μmol m^−2^ s^−1^. Homozygous T-DNA plants were selected based on PCR and harvested for seeds for subsequent experiments. The location of the T-DNA insertion was confirmed via sequencing, and abolition of *WRKY22* expression was tested with RT-qPCR (Supplementary Table S1 at *JXB* online). Expression of *WRKY22* in the TRANSPLANTA-inducible overexpression lines (Coego *et al.*, 2014) was measured with RT-qPCR 24h after application of 10 μM oestradiol in water to the plant trays (Supplementary Table S1). Green peach aphids, *M. persicae*, were reared on radish, *Raphanus sativus* (L.), at 19 °C, 50–70% relative humidity and a 16 h–8h light–dark photoperiod.

### Automated video tracking

Aphid behaviour was tracked on 344 natural accessions of Arabidopsis (*n*=5–6 per accession) according to the methodology of Kloth *et al.* (2015). One adult, wingless aphid was introduced into a well of a 96-well plate containing a leaf disc of 6mm diameter, abaxial side up, on 1% agar substrate. Wells were covered with cling film to avoid aphid escape, and 20 aphids were recorded on 20 different accessions simultaneously with a camera mounted above the plate, at 22±1 °C. EthoVision^®^ XT 8.5 video tracking and analysis software (Noldus Information Technology bv, Wageningen, The Netherlands) was used for automated acquisition of aphid position and velocity. The number and duration of probes were subsequently calculated with the statistical computing program R (R Core Team, 2013). Leaf discs were made of intermediately aged leaves of 4- to 5-week-old Arabidopsis plants, one disc per plant. Aphid behaviour was recorded for 85min, starting at 4.5h after inoculation of the aphids. The video-tracking assay was performed in an incomplete block design with each complete replicate consisting of 18 blocks of 20 accessions. Sixty plants were screened each day across three blocks, and one replicate of the complete Hapmap collection was acquired in 6 days. An alpha design was generated with Gendex (http://designcomputing.net/gendex/) to assign accessions to each block. Five to six replicates were acquired per accession.

### GWA mapping and haplotype analysis

GWA mapping was performed on the proportion of aphids making long probes (> 25min) with scan_GLS (Kruijer *et al.*, 2015), using a kinship matrix based on all SNPs to account for population structure. SNPs with a minor allele frequency <0.05 were excluded from analysis. Block and replicate were included in the model as covariates. SNPs with −log_10_(*P*) value larger than 4 were taken as candidate loci. Generalized heritability was estimated as in Oakey *et al.* (2006). For haplotype analysis, SNPs with a minor allele frequency above 5% were retrieved from the Arabidopsis 1001 genomes browser for 173 accessions (Cao *et al.*, 2011). For each domain, haplotypes were defined as unique SNP combinations with a frequency above 5%. For exons, only non-synonymous SNPs were included. A promoter region of 1000kb was used and gene domains were obtained from Interpro (Mitchell *et al.*, 2015). Promoter motifs were retrieved from Athamap (Hehl and Bülow, 2014).

### RT-qPCR

For each sample, two intermediately aged leaves per 4- to 5-week-old Arabidopsis plant were harvested between 12.00 and 15.00h. Samples were immediately frozen in liquid nitrogen, and stored at −80 °C until processing. RNA was isolated from homogenized leaf material with an InviTrap^®^ Spin Plant RNA kit, and treated with Ambion^®^ TURBO DNA-free^TM^ according to the manufacturer’s instructions. RNA was quantified with a NanoDrop^®^ ND-1 000 spectrophotometer, and integrity was assessed with gel electrophoresis. DNA-free RNA was converted into cDNA using the Bio-Rad iScript^TM^ cDNA synthesis kit. Quantitative reverse transcription PCR was carried out on a Bio-Rad IQ^TM^5 system using SYBR Green. For each primer combination (Supplementary Table S1), RT-qPCR products were sequenced to validate the region of amplification. To test aphid induction of *WRKY22*, *PR1*, *VSP2*, and *PDF1.2*, plants were treated with and without aphids (*n*=4). For infested samples, a Petri dish with indentation for the petiole was used to contain 15 adult *M. persicae* aphids on the leaf, to inflict as little mechanostimulation as possible. Four biological replicates were collected for three treatments: (1) an empty Petri dish for 48h, (2) a Petri dish for 48h with addition of aphids in the last 6h, and (3) a Petri dish with aphids for 48h.

### Electrical penetration graph recording

Feeding behaviour of *M. persicae* aphids was investigated with electrical penetration graph (EPG) recording on 4- to 5-week-old Arabidopsis plants, using direct current (DC) according to the methodology of ten Broeke *et al.* (2013). To adjust the radish-reared aphids to Arabidopsis, aphids were transferred to Col-0 Arabidopsis plants 24h before the experiments. EPG recording was performed at 22±2 °C and light intensity of 120 μmol m^−2^ s^−1^, using clean plants and one aphid per plant. An electrode was inserted in the potting soil and a thin gold wire of 1.5cm was gently attached to the dorsum of an adult, wingless aphid with silver glue. The electrical circuit was completed when the aphid’s piercing–sucking stylet mouthparts penetrated the plant cuticle. Electrical signals associated with stylet activities were recorded and annotated with EPG Stylet+ software (http://www.epgsystems.eu) and further processed in R (R Core Team, 2013; Tjallingii, 1988). Between 20 and 24 biological replicates were measured on T-DNA lines (Col-0: *n*=24; *wrky22-3*: *n*=22; *wrky22-4*: *n*=20) and between 15 and 19 on overexpression lines (Col-0: *n*=15; OE.c: *n*=19; OE.e: *n*=19). *WRKY22* overexpression was induced by supplying 10 μM oestradiol solution to the plants 24h before the experiment. To correct for potential side-effects of oestradiol, the wild-type plants received the same oestradiol treatment as the overexpression lines.

### Aphid population development

To assess aphid developmental rate and population size, 2.5-week-old Arabidopsis plants were infested with one *M. persicae* neonate of age 0–24h and placed in a climate room at 24±1 °C, 50–70% relative humidity, 8 h–16h light–dark photoperiod, 200 μmol m^−2^ s^−1^ light intensity. A soap-diluted water barrier prevented aphids from moving between plants. None of the aphids developed wings. From day 7 onwards, occurrence of the first offspring was checked twice per day using 5× magnification glasses (Col-0: *n*=18; *wrky22-3*: *n*=19; *wrky22-4*: *n*=22). The number of aphids per plant was counted at 14 days after infestation. Plants without an adult aphid 8 days after introduction and plants without any adults or neonates 14 days after introduction were excluded from the analysis.

### Statistics

Data were tested for a normal distribution and homogeneity of variances using Shapiro’s test and Levene’s test. Non-parametric data sets were assessed with the Mann–Whitney *U*-test (two groups) or Kruskall–Wallis test (more than two groups). Data sets with a normal distribution were tested with Student’s *t*-test (two groups) or a one-way ANOVA (more than two groups).

### RNA-seq analysis

RNA-seq analysis was conducted on leaves of Col-0 and *wrky22-3* with three biological replicates per treatment. Five leaves of five different 4- to 5-week-old plants were pooled per sample. Plants had been exposed to one of three treatments: (1) an empty clip cage for 48h, (2) a clip cage for 48h with addition of 15 aphids in the last 6h, and (3) a clip cage with 15 aphids for 48h. Only fourth-instar nymphs and adult *M. persicae* aphids were used. Experiments were conducted simultaneously in a climate chamber (24±1 °C, 50–70% relative humidity, 8 h–16h light–dark photoperiod, and a light intensity of 120 μmol m^−2^ s^−1^), but in separate cages with an air circulation system that prevented contamination of plant volatiles between treatments (Menzel *et al.*, 2014). Samples were harvested in two batches between 13.00 and 16.00h, immediately frozen in liquid nitrogen, and stored at −80 °C until processing. RNA was isolated and checked according to the description above (260/280 OD range: 2.0–2.2, 260/230 OD range: 1.9–2.3). Library preparation was performed with a TruSeq™ RNA Sample Prep Kit (Illumina^®^) and between 11 million and 24 million single-end 50-bp reads were sequenced per sample with Illumina^®^ HiSeq^TM^ 2000 in three lanes, multiplexed with 12 samples per lane. Reads were cleaned from adaptors and trimmed to 51bp using the program Trimmomatic version 0.32 (Bolger *et al.*, 2014). Quality control was performed with FastQC (http://www.bioinformatics.bbsrc.ac.uk/projects/fastqc). Reads were mapped to the TAIR10 Arabidopsis reference genome (https://www.arabidopsis.org/) with Tophat version 2.0.13, intron length 20–2000 (Trapnell *et al.*, 2012; Trapnell *et al.*, 2013). An index file was built with Bowtie 2 (Langmead *et al.*, 2009). Transcript assembly, quantification, normalization and differential expression analysis were performed with Cufflinks, using the bias detection and correction algorithm, multi-read correction for reads mapping to multiple locations, and a minimum alignment count of 10. Treatments were compared both between plant lines (Col-0 versus mutant) and within plant line (empty clip cage versus 6h post-inoculation (hpi), empty clip cage versus 48 hpi, and 6 hpi versus 48 hpi). Only differentially expressed genes (false discovery rate *Q*-value<0.05) with an absolute fold change ≥2 (log2≥1) were taken into account. Differentially expressed genes were tested for overrepresentation of biological processes against a reference set including all transcripts in the complete data set with at least 1 count, using the application BiNGO in Cytoscape (Maere *et al.*, 2005; Cline *et al.*, 2007). Genes associated with cell-wall processes were selected and classified based upon their TAIR description, and the heatmap was constructed with the R package ‘gplots’ (Warnes *et al.*, 2009).

## Results

### GWA mapping

**Fig. 1.**
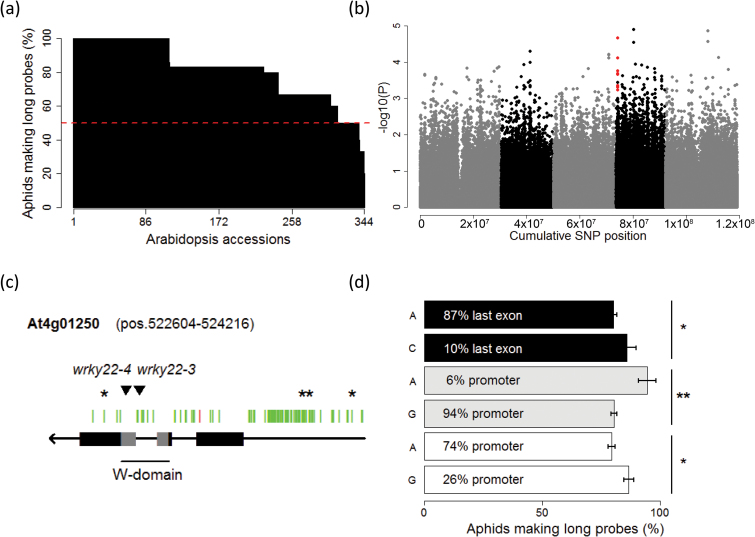
Genome-wide association mapping of aphid feeding behaviour. (A) Phenotypic distribution of the proportion of *M. persicae* aphids making long probes (>25min) during a 1.5h recording on plants from 344 natural Arabidopsis accessions 4.5h post-inoculation. For accessions for which the percentage of long probes was below the dotted line, at least half of the aphids were unsuccessful in feeding. (B) Genome-wide associations with 214 000 SNPs. SNPs in red are positioned in a 40kb region around *WRKY22* (highest −log_10_(*P*)=4.7). (C) All SNPs in *WRKY22* and its 1000kb promoter region according to 173 resequenced Arabidopsis accessions (green: silent; red: non-synonymous). Predicted gene domains are shown in grey, unknown domains in black. Triangles represent T-DNA insertions. (D) One synonymous SNP in the last exon and two SNPs in the promoter had an effect on aphid feeding behaviour (*P<0.05, **P<0.01, Student’s *t*-test, chromosome 4, positions 523037, 524726 and 525079).

To identify genes involved in resistance to the green peach aphid, *M. persicae*, GWA mapping was performed on 344 natural accessions of Arabidopsis, using a selection of approximately 214 000 SNPs (Atwell *et al.*, 2010; Li *et al.*, 2010; Horton *et al.*, 2012). The behaviour of aphids was screened on these accessions with an automated video-tracking platform (Kloth *et al.*, 2015). The number and duration of plant penetrations was estimated by analysing the location and movement of aphids on single leaf discs. It is known that aphids need on average 25min to penetrate the epidermis and mesophyll before they reach the vascular bundle (van Helden and Tjallingii, 1993; Tjallingii, 1994; Prado and Tjallingii, 2007). Therefore, we used the proportion of aphids making long probes (>25min) as a proxy for the success rate of phloem ingestion. The majority of the Arabidopsis accessions did not show indications of resistance to aphids, but on 10% of the accessions at least half of the aphids were unsuccessful in feeding after 4.5h of infestation (Fig. 1A and Supplementary Table S2). GWA mapping of aphid feeding behaviour revealed seven genomic regions with a −log_10_(*P*) value above 4 and a heritability of 10% (Fig. 1B and Table 1). *WRKY22* (*At4g01250*) was identified as a candidate gene in a 40kb region around a polymorphism with a −log_10_(*P*) value of 4.7 (chromosome 4 position 543516). Other candidates in the region included a gene with unknown function (*At4g01290*), a methyltransferase and a gene (*At4g01240*) with an MYB-like domain (*At4g01280*). Resequenced data of 173 accessions (Cao *et al.*, 2011) showed that *WRKY22* contained one non-synonymous SNP in its coding region, and that most of the polymorphisms were confined to the introns and the promoter region (Fig. 1C). A silent SNP in the last exon and two SNPs in the promoter were correlated with aphid feeding behaviour (Fig. 1D). Both polymorphisms in the promoter coincided with an AT-hook DNA-binding motif of AHL20, a transcription factor involved in plant defence to bacteria (Lu *et al.*, 2010). Because WRKY22 is involved in PAMP-triggered immune responses (Asai *et al.*, 2002; Navarro *et al.*, 2004) and its expression is induced by *M. persicae* and *Brevicoryne brassicae* aphids (De Vos *et al.*, 2005; Barah *et al.*, 2013), we conducted further experiments to assess whether WRKY22 is involved in resistance to aphids.

**Table 1.** *SNPs and corresponding genes associated with the proportion of aphids making long probes (>25min,* −*log*_*10*_(P*) value>4*) Only the highest scoring SNP is shown per gene. Genes were grouped in one linkage disequilibrium (LD) region, if they were located within 20kb from each other. Chr.: chromosome.

LD region	Chr.	Position	−log_10_(*P*)	AGI code	Description
1	1	28995670	6.6	*At1g77160*	Protein of unknown function (DUF506)
2	2	10866313	4.3	*At2g25530*	AFG1-like ATPase family protein
3	3	20709836	4.2	*At3g55800*	Chloroplast enzyme sedoheptulose-1,7-bisphosphatase (SBPase)
4	4	519513	4.1	*At4g01240*	*S*-Adenosyl-l-methionine-dependent methyltransferase superfamily protein
4	4	536493	4.1	*At4g01280*	Homeodomain-like superfamily protein, SANT DNA-binding MYB-like domain
4	4	543516	4.7	*At4g01290*	Unknown protein
5	4	6641192	4.5	*At4g10790*	UBX domain-containing protein
5	4	6644022	4.9	*At4g10800*	BTB/POZ domain-containing protein
6	5	15927540	4.9	*At5g39770*	Pseudogene homologous to AtMSU81, restriction Endonuclease
7	5	19854700	4.1	*At5g48965*	Mutator-like transposase family
7	5	19858466	4.1	*At5g48970*	Mitochondrial substrate carrier family protein

### Mesophyll-located susceptibility to aphids

**Fig. 2.**
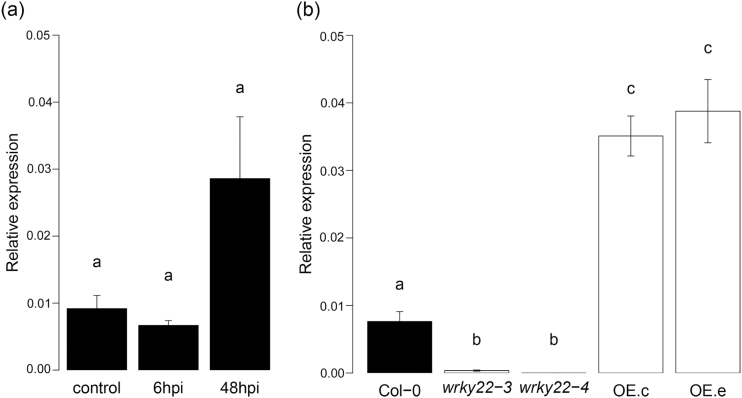
*WRKY22* expression. (A) *WRKY22* expression in the wild-type without *M. persicae* aphids (control) and after 6 and 48h of aphid infestation. (B) Expression in the wild-type (Col-0), *wrky22-3* and *wrky22-4* knockout lines, and *WRKY22*-inducible overexpression lines OE.c and OE.e. Overexpression lines were induced with oestradiol 24h before sampling (one-way ANOVA and Student’s *t*-test; different letters refer to significant differences).

**Fig. 3.**
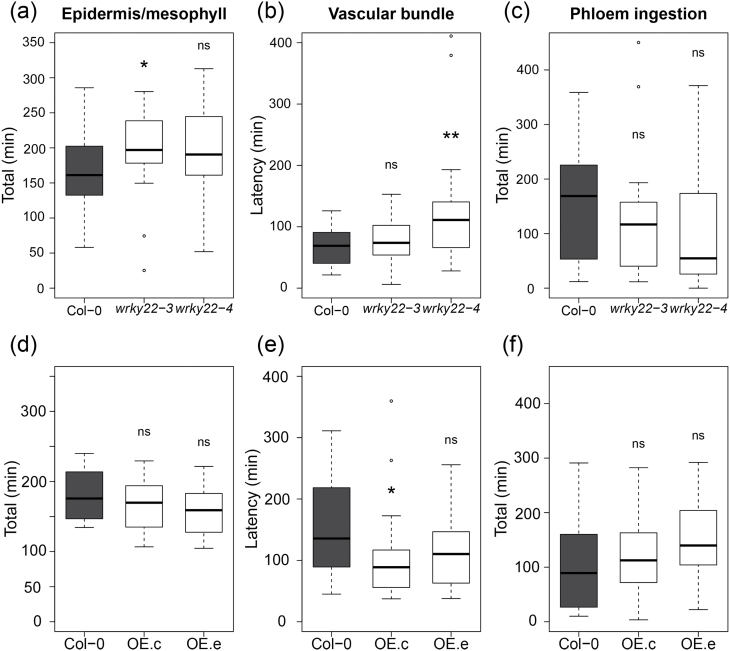
Aphid behaviour on *wrky22* knockout lines (upper panels) and *WRKY22* overexpression lines (lower panels). (A, D) The total time *M. persicae* aphids were penetrating the epidermis and mesophyll during 8-h recordings on knockout lines *wrky22-3* and *wrky22-4* (A), and overexpression lines OE.c and OE.e (D). (B, E) Time between the start of the recording and the first contact with either a phloem or xylem bundle measured on knockout (B), and overexpression lines (E). (C, F) The total time aphids were ingesting phloem on knockout (C), and overexpression lines (F); knockout and overexpression lines were compared with the wild-type with Mann–Whitney *U*-test (*P<0.05; **P<0.01). To test the effect of overexpression, all plants were induced with oestradiol 24h before the assay.

**Fig. 4.**
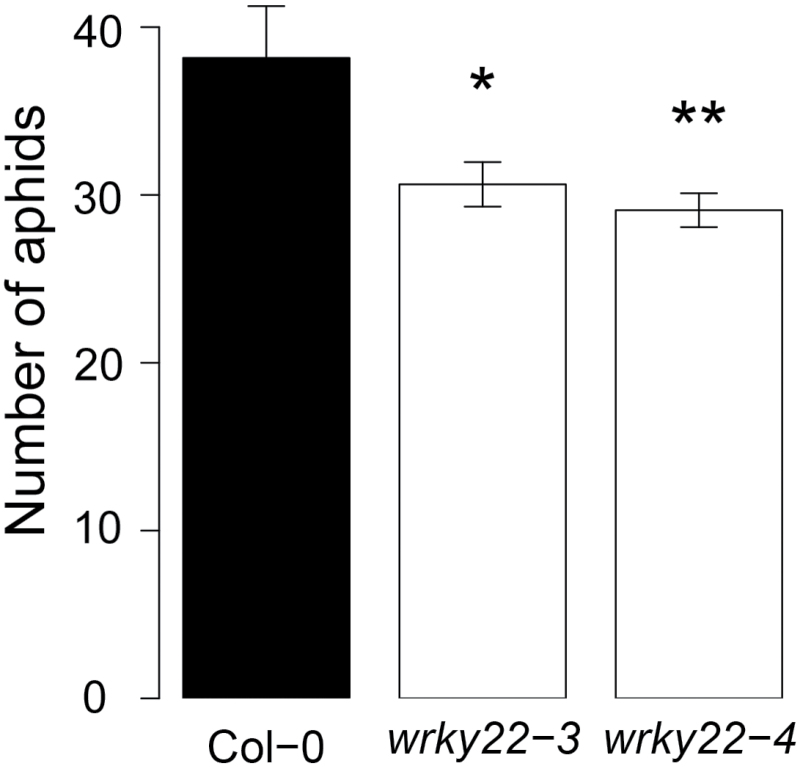
Aphid population size on wild-type and knockout plants. The total number of *M. persicae* aphids per plant was counted 2 weeks after infestation with one neonate aphid. Mutant lines were compared with the wild-type with Student’s *t*-test (*P<0.05; **P<0.01).

To validate the previously reported induction of *WRKY22* by aphid infestation (De Vos *et al.*, 2005; Barah *et al.*, 2013), RT-qPCR was performed on wild-type plants with aphids and without aphids. *WRKY22* expression was unaffected at 6h post-infestation (hpi), and showed a non-significant increase at 48 hpi (Fig. 2A). Two *wrky22* transfer (T)-DNA insertion lines and two *WRKY22*-inducible overexpression lines (Coego *et al.*, 2014) were selected for further experiments. RT-qPCR confirmed that both T-DNA lines were true knockouts, and that the overexpression lines showed a 3- to 5-fold up-regulation of *WRKY22* at 24h after induction with oestradiol (Fig. 2B). For a detailed insight into aphid feeding behaviour on knockout and overexpression lines, we used electrical penetration graph (EPG) recordings (McLean and Kinsey, 1964; Tjallingii, 1988). Aphid feeding behaviour was affected on both *wrky22* knockout lines; on *wrky22-3* aphids spent almost 20% more time on penetrating the epidermis and mesophyll, and on *wrky22-4* aphids showed an hour’s delay in reaching the vascular bundle compared with the wild-type (Col-0) (Fig. 3A–C and Supplementary Table S3). One of the *WRKY22*-inducible overexpression lines showed the opposite trend, with aphids arriving almost an hour earlier at the vascular bundle compared with the wild-type (Fig. 3D–F and Supplementary Table S4). The other *WRKY22* overexpression line did not show any differences compared with the wild-type. The total time of phloem ingestion was not affected in any of the (mutant) lines, suggesting that in the first 8h of infestation, the overall effects are small and confined to activities in the epidermis and/or mesophyll. An aphid population development assay on *wrky22-3* and *wrky22-4* showed that after 2 weeks of infestation, aphid populations were approximately 20% smaller on the knockouts compared with the wild-type (Fig. 4). Both behavioural experiments and population assays indicate that WRKY22 increases susceptibility to *M. persicae* aphids.

### Transcriptomic signature of *wrky22-3*

**Fig. 5.**
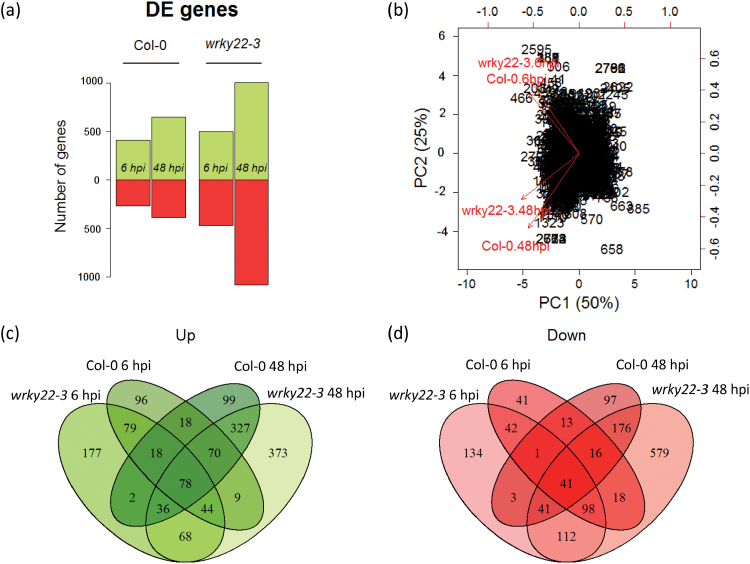
Differentially expressed (DE) genes between treatments with and without aphids in the wild-type and *wrky22-3*. (A) The number of DE genes between control and infestation treatments (green bars: up-regulated, red bars: down-regulated). (B) Biplot of the two first principal components of differentially expressed genes between control and infestation treatments (DE genes ≥2-fold). (C) Overlap in up-regulated genes, and (D) down-regulated genes.

**Fig. 6.**
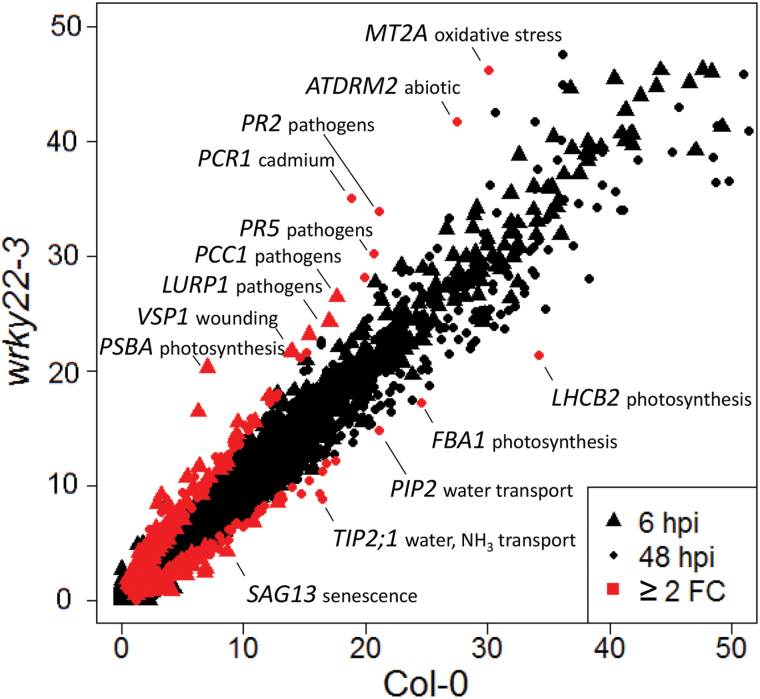
Gene transcripts of aphid-infested wild-type and *wrky22-3* plants. Differentially expressed genes between wild-type and knockout plants (≥2-fold change) are shown in red. Axes depict the square-root transformation of the normalized number of transcripts (the number of fragments per kilobase of transcript per million reads mapped (FPKM)); genes ≤2500 FPKM are shown (including all DE genes in the dataset). Annotations include gene name and biological process; wounding: wound responsive; pathogens: pathogen responsive; cadmium: responsive to cadmium; abiotic: responsive to several abiotic stresses.

**Fig. 7.**
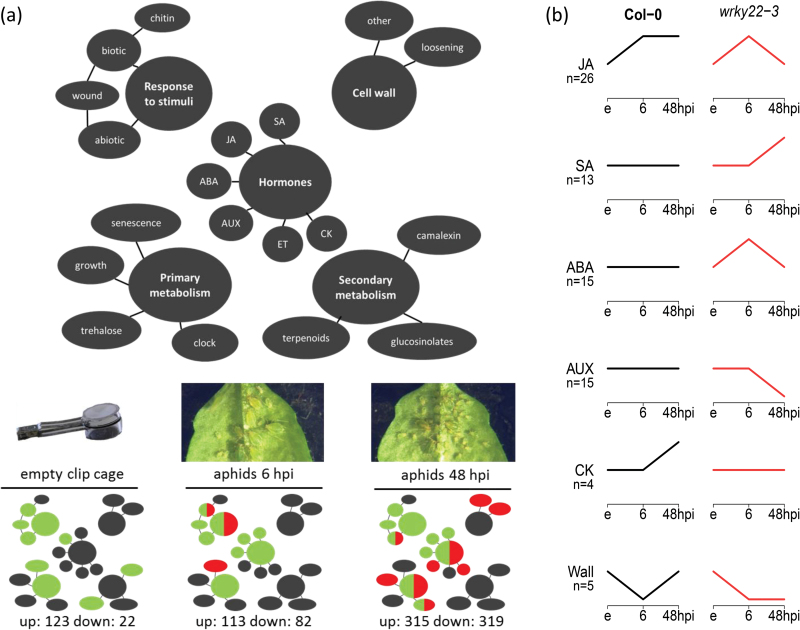
Enriched biological processes in *wrky22-3*. (A) Over-representation of biological processes in the knockout relative to the wild-type. Balloons refer to a process, or to the biosynthesis of, or responsiveness to the respective compound (SA: salicylic acid; JA: jasmonic acid; ABA: abscisic acid; AUX: auxin; ET: ethylene; CK: cytokinin; clock: circadian clock). Balloon colour indicates enrichment in the knockout (green: up-regulated; red: down-regulated; green/red: both up- and down-regulated; the total number of DE genes is depicted below the charts). (B) Relative expression patterns between treatments within each plant line. Only the dominant pattern (≥50% of the genes) of significant perturbations (≥2-fold, q-value <0.05) between treatments with and without aphids is shown. (Wall: cell wall loosening; n: number of genes associated with the biological process; e: empty clip cage; 6: 6 hpi.)

To study the role of *WRKY22* in resistance to aphids, mRNA sequencing (RNA-seq) analysis was performed on *wrky22-3* and wild-type plants that had received one of three treatments: (1) an empty clip cage for 48h, (2) a clip cage for 48h with addition of aphids in the last 6h, and (3) a clip cage with aphids for 48h. For each treatment three biological replicates were sampled, each consisting of a pool of five leaves from different plants. Samples were sequenced for single end 50-bp reads, and for each sample at least 9.5 million reads mapped to unique loci on the Arabidopsis reference genome (Supplementary data file S1). The total number of differentially expressed (DE) genes increased with the duration of infestation, from approximately 700 at 6 hpi to 1000 at 48 hpi in the wild-type. In both treatments, *wrky22-3* contained twice as many up- and down-regulated genes as the wild-type (Fig. 5A). Principal component analysis showed that the duration of infestation was the major factor explaining differential expression (Fig. 5B–D). Highly abundant transcripts that were up-regulated in *wrky22-3* compared with the wild-type included the JA reporter *VSP1* (6 hpi) and pathogenesis-related genes such as *PR2* and *PR5* (48 hpi) (Fig. 6). Photosynthesis- and water-transport-related genes were down-regulated in *wrky22-3* at 48 hpi (Fig. 6). Gene ontology (GO) enrichment analysis of the total set of DE genes revealed an overrepresentation of up-regulated JA-, SA- and abscisic acid (ABA)-responsive genes in *wrky22-3* at both 6 and 48 hpi (Fig. 7A). The JA pathway was mainly characterized by up-regulation of genes of the ethylene response factor (ERF) branch (Vos *et al.*, 2015), e.g. the AP2/ERF transcription factor *RAP2.6*, and *PDF1.2* (Table 2). The majority of the DE genes associated with JA and ABA showed a peak at 6 hpi in *wrky22-3*, whereas most SA-responsive genes reached their highest level at 48 hpi (Fig. 7B). The induction of SA-responsive genes in the T-DNA line at 48 hpi coincided with a suppression of genes associated with auxin (AUX) responsiveness, plant growth and cell wall loosening (Fig. 7A, B).

**Table 2.** *Differentially expressed genes (≥2-fold) of over-represented biological processes in* wrky22-3 *relative to the wild type* GO enrichment and gene classification are according to the BiNGO Cytoscape app (SA: salicylic acid; JA: jasmonic acid; ABA: abscisic acid; AUX: auxin; ET: ethylene; CK: cytokinin) (Cline *et al.*, 2007; Maere *et al.*, 2005).

Process	Treatment	Direction	Name	AGI code	Description
ABA	Empty cage, 6 hpi	Up	*ANNAT4*	*At2g38750*	Annexin, Golgi-mediated secretion
	6 hpi	Up	*HAI1*	*At5g59220*	*HIGHLY ABA-INDUCED PP2C gene 1*
	6 hpi	Up	*HD-Zip-I*	*At3g61890*	Homeodomain leucine zipper class I
	48 hpi	Up	*ACR8*	*At1g12420*	*ACT DOMAIN REPEAT 8*
	48 hpi	Up	*AMY1*	*At4g25 000*	*ALPHA-AMYLASE-LIKE 1*, starch mobilization
	48 hpi	Up	Dehydrins	*At3g50970, At1g20440*	Membrane located, freeze tolerance
	48 hpi	Up	*ERF48*	*At2g40340*	ABA responsive AP2/ERF transcription factor
	48 hpi	Up	*LTI78*	*At5g52310*	*LOW-TEMPERATURE-INDUCED 78*
	48 hpi	Up	*WRKY63*	*At1g66600*	ABA responsive WRKY transcription factor
AUX	6, 48 hpi	Down	*CCA1*	*At2g46830*	Negative regulator of circadian rhythm
	48 hpi	Down	*AXR3*	*At1g04250*	*AUXIN RESISTANT 3*
	48 hpi	Down	*GH3*s	*At2g47750, At5g13360*	*GH3* auxin responsive gene family
	48 hpi	Down	*SAUR*s	*At1g20470, At1g29500, At1g29510, At3g03820, At4g22620, At4g38840, At4g38850, At4g38860, At5g18020, At5g18030, At5g18050*	SAUR(-like) auxin-responsive proteins
CK	48 hpi	Down	*ARR*s	*At1g19050, At1g74890, At3g57040, At5g62920*	Arabidopsis response regulator (ARR) family
JA	Empty cage, 6, 48 hpi	Up	*JAZ*s	*At2g34600, At5g13220, At1g17380, At1g19180*	JAZ7, JAZ10, JAZ5, JAZ1, Jasmonate-Zim- domain proteins
	Empty cage, 6, 48 hpi	Up	*MDHAR4*	*At3g09940*	Monodehydroascorbate reductase
	Empty cage, 6, 48 hpi	Up	*MYB47*	*At1g18710*	JA-responsive MYB transcription factor
	Empty cage, 6, 48 hpi	Up	*TAT3*	*At2g24850*	Tyrosine aminotransferase, JA responsive
	Empty cage	Up	*VSP2*	*At5g24770*	*VEGETATIVE STORAGE PROTEIN 2*
	Empty cage, 6, 48 hpi	Up	*VSP1*	*At5g24780*	*VEGETATIVE STORAGE PROTEIN 1*
	Empty cage, 6 hpi	Up	*AOC*s	*At3g25760, At3g25780*	Allene Oxide Cyclase family, JA biosynthesis
	Empty cage, 6 hpi	Up	*OPR3*	*At2g06050*	*OXOPHYTODIENOATE-REDUCTASE 3*, JA biosynthesis
	Empty cage	Up	*EXT4*	*At1g76930*	Extensin
	Empty cage	Up	*JR1*	*At3g16470*	*JASMONAtE RESPONSIVE 1*
	6, 48 hpi	Up	*PDF1.2*	*At5g44420*	*PLANT DEFENSIN 1.2*
	6 hpi	Up	*DAD1*	*At2g44810*	*DEFECTIVE ANTHER DEHISCENCE 1*, JA biosynthesis
	6 hpi	Up	*JAR1*	*At2g46370*	Jasmonate-amido synthetase
	6 hpi	Up	*LOX3*	*At1g17420*	*LIPOXYGENASE 3*
	6 hpi	Up	*RAP2.6*	*At1g43160*	AP2/ERF transcription factor
SA	6, 48 hpi	Up	*GRX480*	*At1g28480*	Glutaredoxin family, suppresses PDF1.2
	6, 48 hpi	Up	*LURP1*	*At2g14560*	Resistance to *Hyaloperonospora parasitica*
	6, 48 hpi	Up	*WRKY18*	*At4g31800*	*WRKY18*
	48 hpi	Up	*WRKY*s	*At5g01900, At5g22570*	*WRKY38*, *WRKY62*
	48 hpi	Up	*MYB77*	*At3g50060*	*MYB77*
	48 hpi	Up	*WAK1*	*At1g21250*	*CELL WALL-ASSOCIAtED KINASE 1*
JA, SA, ABA	6 hpi	Up	*CIR1*	*At5g37260*	MYB transcription factor
	48 hpi	Up	*MYB*s	*At1g06180, At1g57560, At5g67300, At2g16720*	*MYB13*, *MYB50*, *MYB44*, *MYB7*
	48 hpi	Up	*MPK11*	*At1g01560*	*MAP KINASE 11*
	48 hpi	Up	*PDR12*	*At1g15520*	ABC transporter family, MAPK cascade
Camalexin	Empty cage, 48 hpi	Up	*PAD3*	*At3g26830*	*PHYTOALEXIN DEFICIENT 3*, camalexin biosynthesis
	48 hpi	Up	*P450*	*At4g39950*	Cytochrome P450, indo-3-acetaldoxime (IAOx) biosynthesis
Terpenoids	Empty cage, 6, 48 hpi	Up	*TSP4*	*At1g61120*	*TERPENE SYNTHASE 4*
	Empty cage	Up	*TPS10*	*At2g24210*	*TERPENE SYNTHASE 10*
Cell wall	48 hpi	Down	Expansins	*At1g20190, At1g26770, At1g69530, At2g20750, At2g40610*	Expansin family, cell wall loosening and multidimensional cell growth

### Enhanced negative JA–SA crosstalk in *wrky22-3*

Upon aphid infestation, the *wrky22-3* transcriptome showed evidence of initial suppression, but eventual up-regulation of SA signalling. The expression of *PR1*, a robust SA-reporter gene (Pieterse *et al.*, 2012), was 2-fold down-regulated at 6 hpi, but 3.5-fold up-regulated at 48 hpi in *wrky22-3* compared with the wild-type. Transcript levels of JA-reporter genes *PDF1.2* and *VSP1* were consistently more abundant in *wrky22-3* (Table 3), suggesting a possible role of negative JA–SA crosstalk (Spoel and Dong, 2008; Pieterse *et al.*, 2012). A potential antagonizing candidate is *NIMIN-2*, encoding an SA-suppressing protein (Weigel *et al.*, 2005), which was up-regulated in *wrky22-3* 6 hpi (Table 3). Apart from SA antagonism, there were also signs of JA antagonism. Several up-regulated genes in *wrky22-3*, i.e. *GRX480*, *WRKY51*, and *WRKY62* (Table 3), have previously been implicated as potential suppressors of JA signalling (Mao *et al.*, 2007; Ndamukong *et al.*, 2007; Gao *et al.*, 2011).

**Table 3.** *JA- and SA-signalling-related gene expression in* wrky22-3 *plants compared with wild-type plants with and without aphids* Differentially expressed genes with at least 2-fold absolute change are shown (ns: not significant; emp: empty clip cage; SA/JA sig: SA/JA signalling; SA/JA suppr: suppression of SA/JA signalling).

			Fold change			
Gene	Name	Role	Emp	6 hpi	48 hpi	Reference
*VSP1*	*VEGETATIVE STORAGE PROTEIN 1*	JA sig	13.0	2.5	2.8	(Anderson *et al.*, 2004; Lorenzo *et al.*, 2004)
*VSP2*	*VEGETATIVE STORAGE PROTEIN 2*	JA sig	7.5	ns	ns	(Anderson *et al.*, 2004; Lorenzo *et al.*, 2004)
*PDF1.2, 1.2C*	*PLANT DEFENSIN 1.2A, 1.2C*	JA sig	ns	2.3	3.5	(Lorenzo *et al.*, 2003; Penninckx *et al.*, 1998)
*PR1*	*PATHOGENESIS-RELATED GENE 1*	SA sig	ns	0.4	3.5	(van Loon *et al.*, 2006)
*NIMIN-1*	*NIM1-INTERACTING 1*	SA suppr	ns	ns	2.5	(Weigel *et al.*, 2001; Weigel *et al.*, 2005)
*NIMIN-2*	*NIM1-INTERACTING 2*	SA suppr	ns	2.0	2.5	(Weigel *et al.*, 2001; Weigel *et al.*, 2005)
*GRX480*	Glutaredoxin	JA suppr	ns	2.8	2.8	(Ndamukong *et al.*, 2007)
*WRKY51*	*WRKY DNA-BINDING PROTEIN 51*	JA suppr	ns	ns	4.3	(Gao *et al.*, 2011)
*WRKY62*	*WRKY DNA-BINDING PROTEIN 62*	JA suppr	ns	ns	2.8	(Mao *et al.*, 2007)

### JA induction by mechanostimulation in *wrky22-3*

**Fig. 8.**
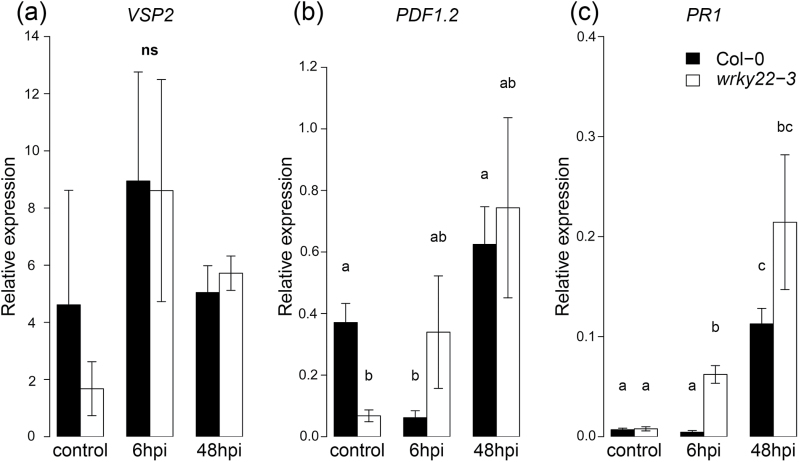
The effect of aphid infestation without clip cage on the expression of JA and SA reporter genes. RT-qPCR measurements of expression of the JA reporters *VSP2* (A) and *PDF1.2* (B), and the SA reporter *PR1* (C) in wild-type and *wrky22-3* plants (Student’s *t*-test and the Mann–Whitney *U*-test; different letters denote significant differences). Aphids were contained on the leaves without inflicting major mechanical stimulation (see Materials and methods).

Remarkably, the treatment with empty clip cages changed the expression of almost 150 genes in the knockout relative to the wild-type. Most of them were up-regulated in *wrky22-3* and showed a significant overrepresentation of JA-responsive genes, including *VSP2* of the touch- and wound-responsive MYC-branch of the JA signalling pathway (Lange and Lange, 2015) (Fig. 7 and Table 2). In order to see if mechanostimulation by clip cages had affected the plant’s response to aphids, RT-qPCR was conducted on aphid-infested leaves without clip cages (see Materials and methods). We found that in clean *wrky22-3* plants, *PDF1.2* expression was lower compared with wild-type plants (Fig. 8). After 6h of aphid infestation, *PR1* was up-regulated in *wrky22*-3 while *PDF1.2* only showed a non-significant increase (Fig. 8). These results suggest that, in the RNA-seq analysis, the over-represented JA response may have been clip cage-induced and was likely involved in the suppression of the SA response to aphids at 6 hpi.

### Known resistance genes

Several genes described in earlier studies as being involved in enhancing resistance to *M. persicae* were up-regulated in *wrky22-3*. Expression of the MYB-domain-containing protein MYBR1, found to affect *M. persicae* reproduction under the influence of harpin proteins (Liu *et al.*, 2010), was more than 2-fold induced at 48 hpi (Supplementary data file S1). Also several other genes with affiliation to known resistance factors for *M. persicae* were up-regulated, such as the phloem protein PP2-A12 at 48 hpi, the myrosinase-binding protein MBP1 at 48 hpi, and several xyloglucan endotransglucosylases/hydrolases (XTHs) at 6 and 48 hpi (Mewis *et al.*, 2005; Divol *et al.*, 2007; Zhang *et al.*, 2011; Louis and Shah, 2013).

### Differential expression of cell wall-related genes

**Fig. 9.**
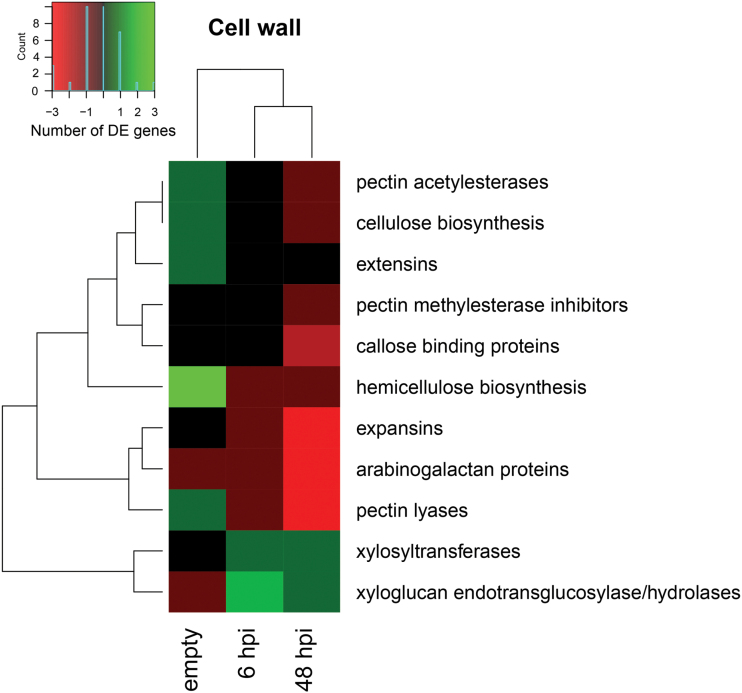
Expression of cell-wall related genes in *wrky22-3* and the effect of mechanostimulation by empty clip cages and aphid infestation. Genes and treatments are clustered according to the number of differentially expressed genes (≥2-fold change), using Ward’s minimum variance method (red: down-regulated; green: up-regulated in *wrky22-3* compared with the wild-type).

After 48h of aphid infestation, the *wrky22-3* transcriptome was characterized by down-regulation of genes associated with cell wall loosening (Fig. 7 and Table 2). To assess all cell wall-related processes, DE genes with cell wall annotation were selected and grouped into categories based on their name and function (Fig. 9). We did not observe an up-regulation of touch-responsive xyloglucan endotransglucosylases/hydrolases (XTHs) by the empty clip cage. The empty clip cage did, however, cause a 3- to 5-fold up-regulation of the cellulose synthase-like genes *CSLA1*, *CSLA10*, and *CSLA15*, involved in hemicellulose biosynthesis (Liepman *et al.*, 2005). Aphids up-regulated XTHs and down-regulated, for example, expansins, involved in cell-wall loosening, and pectin lyases, involved in pectin breakdown. While cell-wall loosening is a prerequisite for cell elongation, a process mainly regulated by CK and AUX (Taiz, 1984; Yadav *et al.*, 2009; Albersheim *et al.*, 2011), the transcriptomic patterns indicate an aphid-induced arrest of symplastic cell growth in *wrky22-3*.

## Discussion

### The effect of WRKY22 on *M. persicae* aphids

Plant responses to aphids are known to involve many transcriptional perturbations including multiple phytohormonal pathways (De Vos *et al.*, 2005; De Vos *et al.*, 2007; Smith and Boyko, 2007; Foyer *et al.*, 2015). It is, therefore, a challenge to unravel the genetic basis of effective defence mechanisms against aphids. In this study, we explored natural variation in Arabidopsis to find genes related to impaired feeding behaviour of *M. persicae*. Natural variation in the occurrence of long probes, a proxy for the success rate of phloem ingestion, was associated to several genomic regions, including the *WRKY22* locus. Polymorphisms in the *WRKY22* promoter and in the last exon most strongly correlated with variation in aphid feeding behaviour. Even though the associations had low statistical power and heritability, knockout lines confirmed an effect of *WRKY22* on aphid performance. Without a functional WRKY22 protein, it was more difficult for aphids to penetrate the epidermis and mesophyll and they arrived later at the vascular bundle. One *WRKY22* overexpression line showed the opposite trend, although the impact was smaller than in the *wrky22* knockouts, most likely due to the moderate extent and short time frame of the overexpression (3- to 5-fold change, induced 24h before the experiments). The effects of WRKY22 on aphid performance were marginal in the first 8h, but more substantial after an infestation period of 2 weeks as reflected by aphid population size. Overall, these assays indicate that WRKY22 promotes susceptibility to *M. persicae* aphids. Our RNA-seq and RT-qPCR analyses indicate that WRKY22-mediated susceptibility is associated with the suppression of SA signalling. Aphid infestation of *wrky22-3* plants resulted in faster and potentially stronger up-regulation of the SA pathway than in wild-type plants. Even though it has been described that JA-induced defences are most effective against aphids (De Vos *et al.*, 2007; Walling, 2008), SA-induced mechanisms have been shown to have a detrimental impact on aphids as well (Li *et al.*, 2006; Moloi and Westhuizen, 2006). The down-regulation of pectin lyases and expansins (Fig. 9) suggests that there is less degradation of pectin and less loosening of the cell wall matrix in the *wrky22* mutant. Fortification of the primary cell wall may have hampered the penetration of the mesophyll apoplast by aphids. This would explain why aphids required more time in probing the epidermis and mesophyll and were delayed in reaching the vascular bundle on the *wrky22* mutants (Fig. 3 and Supplementary Table S3). We can, however, not exclude the involvement of other resistance factors in the mesophyll, such as the accumulation of reactive oxygen species or secondary metabolites.

### Involvement of WRKY22 in biotic and abiotic stress responses

Apart from *WRKY22*’s responsiveness to aphids, we observed a strong activation of the JA pathway in *wrky22-3* as a result of the use of clip cages. JA accumulation is known to be induced by mechanical stimuli (Ichimura *et al.*, 2000; Chehab *et al.*, 2012), wounding, and damage-associated molecular patterns (DAMPs) (Doares *et al.*, 1995; Denoux *et al.*, 2008; Vidhyasekaran, 2014). Since there were no obvious signs of plant damage, the up-regulation of the JA pathway was most likely triggered by touch-induced surface stimulation of our samples. *WRKY22* has been shown to be induced in response to touch and wounding (Lee *et al.*, 2004; Kilian *et al.*, 2007). Our data suggest that WRKY22 acts as a suppressor of JA signalling in response to these stimuli. Many other abiotic stimuli have been described to induce *WRKY22* as well, such as prolonged darkness, submergence, cold acclimation, light perception, salinity, potassium starvation, and exposure to ozone (Folta *et al.*, 2003; Hampton *et al.*, 2004; Monte *et al.*, 2004; Lee *et al.*, 2005; Tosti *et al.*, 2006; Chawade *et al.*, 2007; Kilian *et al.*, 2007; Zhou *et al.*, 2011; Göhre *et al.*, 2012; Hsu *et al.*, 2013; Kim *et al.*, 2013; Sugimoto *et al.*, 2014). Hsu *et al.* (2013) identified several potential downstream targets of WRKY22, including genes involved in drought resistance and phosphate starvation. The accumulating evidence for its involvement in abiotic stress responses warrants a change of view, i.e. that WRKY22 is not solely involved in PTI. A parallel can be drawn with WRKY40, previously known as a repressor of PTI but recently also recognized as a central player in ABA inhibition during abiotic stress (Chen *et al.*, 2010; Friedel *et al.*, 2012). Although abiotic and biotic stimuli are most likely perceived via stress-specific mechanisms and require differential plant responses, signal-transduction pathways might converge via common regulators, such as WRKY22, in order to fine-tune the interplay between phytohormones.

### SA–JA signal integration

**Fig. 10.**
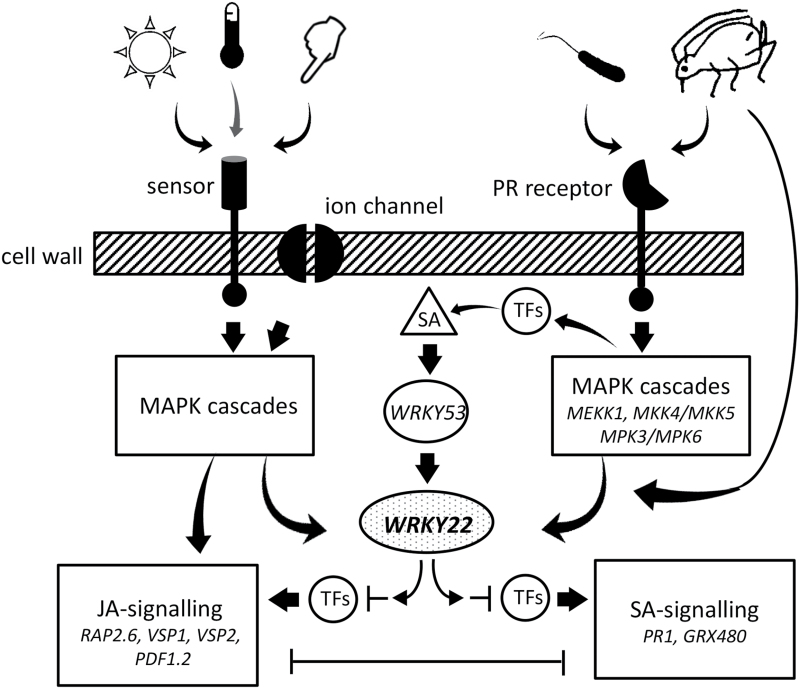
Hypothetical model of WRKY22’s role in plant response to abiotic (left) and biotic (right) stresses. Changes in, for example, light, temperature and touch are perceived via sensors and ion channels; plant invasion by organisms such as bacteria and aphids is mainly perceived via pattern-recognition (PR) receptors. These stimuli induce *WRKY22* directly via MAPK cascades (Ichimura *et al.*, 2000; Asai *et al.*, 2002), or indirectly via SA accumulation (Miao *et al.*, 2004; Miao and Zentgraf, 2007). Alternatively, aphid effectors secreted via the saliva may induce *WRKY22* via PR-receptor-independent routes. WRKY22 subsequently integrates signalling of the JA and SA pathway, by inhibiting or activating specific transcription factors (TFs) and other regulatory genes.

One of the major questions is whether WRKY22 is an activator or repressor of SA and JA signalling. Our transcriptome analysis of *wrky22-3* revealed up-regulation of JA signalling upon mechanostimulation and up-regulation of SA signalling upon aphid infestation. This would suggest that in wild-type plants, WRKY22 is a suppressor of JA and SA signalling. From previous studies we know, however, that WRKY22 and WRKY29 confer resistance to (hemi)biotrophic and necrotrophic pathogens (Asai *et al.*, 2002; Hsu *et al.*, 2013), and that they are induced by PAMP-triggered MAPK cascades which result in the activation of SA, JA and ET signalling (Zipfel *et al.*, 2004). There is no direct evidence that WRKY22 and its homologue WRKY29 induce SA, JA and ET signalling, and the possibility exists that they are involved in MAPK-triggered processes independent of SA and JA signalling. Nevertheless, their requirement for PTI makes them unlikely candidates for consistent suppression of plant defence hormones. Rather, WRKY22 could be an integrator of SA and JA signals, inhibiting or enforcing both pathways, depending on their interaction with other transcription factors and signalling pathways (Fig. 10). Similarly, WRKY70 has been proposed to be capable of inducing and inhibiting both SA and JA signalling, depending on the strength of the induction (Li *et al.*, 2004; Ülker *et al.*, 2007). Although many questions remain with regard to the underlying mechanism, our study shows that WRKY22 plays a role in both SA and JA signalling and is involved in transcriptional reprogramming in response to mechanostimulation and aphid infestation. To understand the function of *WRKY22*, its transcriptional network needs to be further unravelled under multiple biotic and abiotic stress conditions. With respect to aphids, WRKY22 increases susceptibility. Its responsiveness to aphid infestation and its potential to suppress JA and SA signalling would make WRKY22 an excellent target for aphids to manipulate JA- and SA-dependent host plant defences for their own benefit.

## Supplementary data

Supplementary data are available at *JXB* online.

Data file S1. Differentially expressed genes between *wrky22-3* and wild-type rosette leaves with and without aphid infestation.

Table S1. Primers used for PCR and RT-qPCR.

Table S2. Percentage of aphids making long probes (>25min) 4.5 hour after inoculation on 344 natural Arabidopsis accessions.

Table S3. Aphid feeding behaviour, measured by 8-hour EPG recordings on wild-type (Col-0) and *wrky22* T-DNA lines (*wrky22-3* and *wrky22-4*).

Table S4. Aphid feeding behaviour, measured by 8-hour EPG recordings on wild-type (Col-0) and *wrky22*-inducible overexpression lines (OE.c and OE.e).

Supplementary Data
