# Supplementary material for: AtWRKY22 promotes susceptibility to aphids and modulates salicylic acid and jasmonic acid signalling
Source: J Exp Bot. 2016 Apr 23;67(11):3383–96. doi: 10.1093/jxb/erw159 (PMC4892728; doi:10.1093/jxb/erw159)
Supplement: Supplementary Data [file supp_67_11_3383__index.html]

AtWRKY22 promotes susceptibility to aphids and modulates salicylic acid and jasmonic acid signalling — AtWRKY22 promotes susceptibility to aphids and modulates salicylic acid and jasmonic acid signalling — Supplementary Data 

# AtWRKY22 promotes susceptibility to aphids and modulates salicylic acid and jasmonic acid signalling

## Supplementary Data

Data files

- Supplemental\_Tables\_S1\_S4.pdf - Supplementary Data
- Supplemental\_dataset\_S1.xlsx - Supplementary Data
