# Supplementary material for: AtWRKY22 promotes susceptibility to aphids and modulates salicylic acid and jasmonic acid signalling
Source: J Exp Bot. 2016 Apr 23;67(11):3383–96. doi: 10.1093/jxb/erw159 (PMC4892728; doi:10.1093/jxb/erw159)
Supplement: Supplementary Data [file supp_erw159_Supplemental_Tables_S1_S4.pdf]

Table S1. Primers used for PCR and RT-qPCR.

**PCR**			
**Mutant/insertion**	**SALK code**	**Direction**	**Primer**
T-DNA insertion	LBb1.3		ATTTTGCCGATTTCGGAAC
wrky22-3	SALK_094892	Fw	AAACCCTATATTCACCGTCGG
		Rev	ATATTCCTCCGGTGGTAGTGG
wrky22-4	SALK_098205	Fw	CACAGAACCAGAAACGTCCTC
		Rev	ATATTCCTCCGGTGGTAGTGG
**RT-qPCR**			
**Gene name**	**AGI code**	**Direction**	**Primer**
PEX4	*At5g25760*	Fw	TGCAACCTCCTCAAGTTCG
	At5g25760	Rev	CACAGACTGAAGCGTCCAAG
ACT2	*At3g18780*	Fw	CCCGATGGGCAAGTCATCACGAT
	At3g18780	Rev	GTCTCGTGGATTCCAGCAGCTTCC
WRKY22	*At4g01250*	Fw	CATGGCGAAAGTACGGACAG
	At4g01250	Rev	GAATTACGGTGTGTCGGAGC
PDF1.2	*At5G44420*	Fw	TTGCTGCTTTCGACGCA
	At5G44420	Rev	TGTCCCACTTGGCTTCTCG
VSP2	*At5G24770*	Fw	CATAGACTTCGACACGGTGC
	At5G24770	Rev	TTTGCACACGGTTTTGGAGT
PR1	*At2G14610*	Fw	GTTGCAGCCTATGCTCGGAG
	At2G14610	Rev	CCGCTACCCCAGGCTAAGTT

Table S2. Percentage of aphids making long probes (> 25 min) 4.5 hour after inoculation on 344 natural Arabidopsis accessions.

Genotype	Line	%
CS76214	Pro-0	20
CS28163	Co-2	33
CS28808	Wag-3	33
CS76107	CAM-16	33
CS76127	Est-1	33
CS76287	UKSE06-429	33
CS28214	Dra-2	40
CS28091	Boot-1	50
CS28097	Bs-2	50
CS28160	Cnt-1	50
CS28217	Ede-1	50
CS28345	Hh-0	50
CS28419	Kr-0	50
CS28492	Mh-0	50
CS28633	PHW-33	50
CS28635	PHW-35	50
CS28645	Pn-0	50
CS28725	Sav-0	50
CS28758	Tha-1	50
CS28823	Ws	50
CS76098	Blh-1	50
CS76106	C24	50
CS76150	Kas-1	50
CS76152	Kelsterbach-4	50
CS76165	LI-OF-095	50
CS76172	LL-0	50
CS76190	Mr-0	50
CS76196	NC-6	50
CS76208	Paw-3	50
CS76221	ROM-1	50
CS76276	UKNW06-060	50
CS76281	UKSE06-192	50
CS76108	CAM-61	60
CS76109	Can-0	60
CS76129	Fei-0	60
CS76131	Fjä1-2	60
CS76137	Gr-1	60
CS76177	Lp2-6	60
CS76252	TOU-A1-115	60
CS76291	UKSE06-628	60

CS28049	Ann-1	67
CS28053	Ba-1	67
CS28135	Chat-1	67
CS28140	CIBC-2	67
CS28141	CIBC-4	67
CS28252	Fi-1	67
CS28344	Hey-1	67
CS28350	Hn-0	67
CS28373	Jm-1	67
CS28423	Krot-2	67
CS28459	Li-6	67
CS28614	PHW-14	67
CS28628	PHW-28	67
CS28631	PHW-31	67
CS28685	Rhen-1	67
CS28692	Rou-0	67
CS28734	Sh-0	67
CS28739	Si-0	67
CS28787	Uk-1	67
CS28795	Utrecht	67
CS28800	Ven-1	67
CS28804	Wa-1	67
CS28812	WAR	67
CS76088	Alc-0	67
CS76090	ALL1-3	67
CS76093	Bå1-2	67
CS76094	Bay-0	67
CS76097	Bla-1	67
CS76101	Br-0	67
CS76104	BUI	67
CS76105	Bur-0	67
CS76124	Duk	67
CS76128	Fäb-4	67
CS76149	Ka-0	67
CS76159	Lc-0	67
CS76164	Ler-1	67
CS76169	Lis-1	67
CS76171	Lisse	67
CS76174	Lom1-1	67
CS76181	MIB-15	67
CS76195	Na-1	67
CS76200	Ömö2-1	67
CS76202	Ost-0	67
CS76203	Oy-0	67
CS76207	PAR-5	67
CS76226	Se-0	67

CS76227	Shahdara	67
CS76228	SLSP-30	67
CS76231	St-0	67
CS76232	Ste-3	67
CS76233	T1040	67
CS76234	T1060	67
CS76236	T1110	67
CS76244	Tamm-2	67
CS76254	TOU-A1-12	67
CS76257	TOU-A1-67	67
CS76262	TOU-H-13	67
CS76263	TOU-I-17	67
CS76280	UKSE06-062	67
CS76282	UKSE06-272	67
CS76288	UKSE06-466	67
CS76306	Zdr-6	67
CS28108	Bu-8	80
CS28241	Es-0	80
CS28420	Kro-0	80
CS28583	Old-1	80
CS28729	Sei-0	80
CS76086	627ME-4Y1	80
CS76114	Ct-1	80
CS76115	CUR-3	80
CS76157	LAC-3	80
CS76184	MIB-84	80
CS76191	Mrk-0	80
CS76224	Sap-0	80
CS76230	Sq-8	80
CS76251	Tottarp-2	80
CS76259	TOU-C-3	80
CS76293	Ull2-3	80
CS76298	Vår2-1	80
CS28014	Amel-1	83
CS28017	An-2	83
CS28051	Arby-1	83
CS28064	Benk-1	83
CS28090	Blh-2	83
CS28099	Bsch-0	83
CS28165	Co-4	83
CS28193	Com-1	83
CS28200	Da-0	83
CS28243	Est-0	83
CS28268	Fr-4	83
CS28277	Ge-1	83
CS28279	Gel-1	83

CS28364	Je-0	83
CS28369	Jl-3	83
CS28394	Kl-5	83
CS28457	Li-5:2	83
CS28490	Mc-0	83
CS28495	Mnz-0	83
CS28510	N4	83
CS28513	N7	83
CS28580	Ob-1	83
CS28610	PHW-10	83
CS28613	PHW-13	83
CS28620	PHW-20	83
CS28622	PHW-22	83
CS28636	PHW-36	83
CS28637	PHW-37	83
CS28640	Pla-0	83
CS28650	Pog-0	83
CS28743	Sp-0	83
CS28750	Ste-0	83
CS28779	Tscha-1	83
CS28788	Uk-2	83
CS28810	Wag-5	83
CS28814	Wc-2	83
CS28822	Wl-0	83
CS28833	Wt-3	83
CS28848	Ors-1	83
CS28849	Ors-2	83
CS76083	11ME1.32	83
CS76084	11PNA4.101	83
CS76091	An-1	83
CS76095	Belmonte-4-94	83
CS76096	Bg-2	83
CS76099	Bor-1	83
CS76103	Bu-0	83
CS76110	Cen-0	83
CS76113	Col-0	83
CS76122	DraIV	83
CS76125	Eden-2	83
CS76126	Edi-0	83
CS76132	Fjä1-5	83
CS76134	Gd-1	83
CS76138	Gul1-2	83
CS76139	Gy-0	83
CS76140	Hi-0	83
CS76141	Hod	83
CS76144	HR-5	83

CS76145	Hs-0	83
CS76148	JEA	83
CS76151	KBS-Mac-8	83
CS76155	Köln	83
CS76161	LDV-25	83
CS76163	LDV-58	83
CS76168	Lip-0	83
CS76170	Lis-2	83
CS76176	Lp2-2	83
CS76180	Map-42	83
CS76185	MNF-Che-2	83
CS76186	MNF-Jac-32	83
CS76187	MNF-Pot-48	83
CS76188	MNF-Pot-68	83
CS76192	Mt-0	83
CS76193	Mz-0	83
CS76197	Nd-1	83
CS76205	PAR-3	83
CS76209	Pent-1	83
CS76210	Per-1	83
CS76211	Petergof	83
CS76215	Pu2-23	83
CS76216	Ra-0	83
CS76217	Rak-2	83
CS76220	Rmx-A180	83
CS76222	Rsch-4	83
CS76225	Sav-0	83
CS76229	Sparta-1	83
CS76235	T1080	83
CS76239	T540	83
CS76247	TDr-18	83
CS76248	TDr-3	83
CS76253	TOU-A1-116	83
CS76256	TOU-A1-62	83
CS76264	TOU-I-2	83
CS76265	TOU-I-6	83
CS76272	UKID37	83
CS76273	UKID48	83
CS76278	UKNW06-436	83
CS76279	UKNW06-460	83
CS76283	UKSE06-278	83
CS76284	UKSE06-349	83
CS76286	UKSE06-414	83
CS76289	UKSE06-482	83
CS76292	UKSW06-202	83
CS76297	Van-0	83

CS76299	VOU-1	83
CS76300	VOU-2	83
CS76302	Wil-1	83
CS76304	Wt-5	83
CS76305	Yo-0	83
CS76308	ZdrI	83
CS76213	Pna-17	86
CS22689	RRS-10	100
CS28007	Aa-0	100
CS28013	Alst-1	100
CS28018	Ang-0	100
CS28054	Baa-1	100
CS28063	Be-1	100
CS28128	Ca-0	100
CS28133	Cha-0	100
CS28142	CIBC-5	100
CS28158	Cit-0	100
CS28181	CSHL-5	100
CS28201	Da(1)-12	100
CS28202	Db-0	100
CS28208	Di-1	100
CS28210	Do-0	100
CS28236	Ep-0	100
CS28280	Gie-0	100
CS28282	Go-0	100
CS28326	Gr-5	100
CS28332	Gu-1	100
CS28336	Ha-0	100
CS28382	Kelsterbach-2	100
CS28395	Kn-0	100
CS28407	KNO-11	100
CS28454	Li-3	100
CS28461	Li-7	100
CS28527	Nc-1	100
CS28550	NFC-20	100
CS28564	No-0	100
CS28568	Nok-1	100
CS28573	Nw-0	100
CS28575	Nw-2	100
CS28578	Nz1	100
CS28587	Or-0	100
CS28626	PHW-26	100
CS28651	Pr-0	100
CS28663	Pu2-24	100
CS28713	RRS-7	100
CS28720	S96	100

CS28724	Sapporo-0	100
CS28732	Sg-1	100
CS28760	Tiv-1	100
CS28780	Tsu-0	100
CS28786	Ty-0	100
CS28809	Wag-4	100
CS28847	Zu-1	100
CS76085	328PNA054	100
CS76087	Ag-0	100
CS76089	ALL1-2	100
CS76092	App1-16	100
CS76100	Bor-4	100
CS76102	Brö1-6	100
CS76111	CIBC-17	100
CS76116	Cvi-0	100
CS76119	DraIV	100
CS76120	DraIV	100
CS76121	DraIV	100
CS76123	DraIV	100
CS76135	Ge-0	100
CS76136	Got-7	100
CS76142	Hov4-1	100
CS76143	Hovdala-2	100
CS76146	HSm	100
CS76147	In-0	100
CS76153	Kin-0	100
CS76154	Kno-18	100
CS76156	Nordborg	100
CS76158	LAC-5	100
CS76160	LDV-14	100
CS76162	LDV-34	100
CS76166	Liarum	100
CS76167	Lillö-1	100
CS76173	Lm-2	100
CS76175	Löv-5	100
CS76179	Lz-0	100
CS76182	MIB-22	100
CS76183	MIB-28	100
CS76189	MOG-37	100
CS76194	N13	100
CS76198	NFA-10	100
CS76199	NFA-8	100
CS76201	Ör-1	100
CS76206	PAR-4	100
CS76212	PHW-34	100
CS76218	Ren-1	100

CS76219	Rev-2	100
CS76223	Sanna-2	100
CS76238	T510	100
CS76240	T620	100
CS76242	Ta-0	100
CS76243	TÅD	100
CS76245	TDr-1	100
CS76249	TDr-8	100
CS76250	Tomegap-2	100
CS76255	TOU-A1-43	100
CS76258	TOU-A1-96	100
CS76260	TOU-E-11	100
CS76261	TOU-H-12	100
CS76266	TOU-J-3	100
CS76267	TOU-K-3	100
CS76268	Ts-1	100
CS76269	UduI	100
CS76270	UKID101	100
CS76274	UKID80	100
CS76275	UKNW06-059	100
CS76277	UKNW06-386	100
CS76285	UKSE06-351	100
CS76290	UKSE06-520	100
CS76295	Ull3-4	100
CS76296	Uod-7	100
CS76301	Wei-0	100
CS76303	Ws-0	100
CS76307	ZdrI	100

Table S3. Aphid feeding behaviour, measured by 8-hour EPG recordings on wild type (Col-0) and *wrky22* T-DNA lines (*wrky22-3* and *wrky22-4*).

Tissue	Variable	Col-0	*wrky22-3*	*wrky22-4*
Epidermis/mesophyll	Total duration NP (min)	111.6 ± 11.8	105.5 ± 13.6ns	153.1 ± 17.2ns
	Latency to 1st C (min)	6 ± 1.2	7.5 ± 1.8ns	12.4 ± 2.5ns
	Total duration C (min)	167.5 ± 11.2	**197.6 ± 12.8***	192 ± 14.1ns
	Number of C	43.1 ± 4.4	41.4 ± 4.9ns	51.6 ± 5.5ns
	Number of C < 3 min	29.3 ± 4.1	25.5 ± 4.2ns	38 ± 4.9ns
	Number of C < 20 sec	12.1 ± 2.5	8 ± 2.1ns	17.6 ± 2.9ns
	Mean duration C (min)	4.6 ± 0.4	**6.7 ± 1***	4.4 ± 0.4ns
	Potential drops in C (min^{-1})	1.2 ± 0	1.2 ± 0ns	1.2 ± 0.1ns
	Total duration F (min)	2.8 ± 2.8	0 ± 0ns	6.2 ± 4.4ns
	Number of F	0.1 ± 0.1	0.1 ± 0.1ns	0.4 ± 0.2ns
Vascular bundle	Latency to 1st E1 after C (min)	67.8 ± 7.3	74.9 ± 8.1ns	119.8 ± 23.7ns
	Mean latency E1 after C (min)	10.5 ± 0.7	12.7 ± 1.3ns	14.2 ± 1.6ns
	Total duration E1 (min)	10.4 ± 1.3	13.3 ± 1.7ns	7.1 ± 0.6ns
	Number of E1	11.3 ± 1.4	12.5 ± 1.3ns	8.1 ± 0.9ns
	Mean duration E1 (min)	1.1 ± 0.2	1.2 ± 0.2ns	1 ± 0.1ns
	Number of single E1	1.2 ± 0.3	1.4 ± 0.3ns	1.4 ± 0.3ns
	E1 in E (%)	8.4 ± 1.5	10.8 ± 1.7ns	12.7 ± 3.6ns
	Latency to 1st E2 (min)	96.5 ± 12.5	107.3 ± 17.8ns	144.1 ± 24.6ns
	Total duration E2 (min)	168.3 ± 19.9	154 ± 21.3ns	117.9 ± 24.1ns
	Number of E2	8.2 ± 1	9 ± 0.9ns	5.8 ± 0.8ns
	Mean duration E2 (min)	32.5 ± 7	41.8 ± 21ns	24.1 ± 6.9ns
	Total duration E2s (min)	148.8 ± 20.9	129.1 ± 22.9ns	103.5 ± 24.1ns
	Aphids with E2 (%)	100 ± 0	100 ± 0ns	100 ± 0ns
	E2/C ratio	1.3 ± 0.3	1.6 ± 0.8ns	**1 ± 0.4***
	Latency to vascular bundle (min)	69.3 ± 6.5	78.7 ± 8.6ns	**130.2 ± 22.5****
	Total duration G (min)	19.4 ± 6.3	9.5 ± 3.8ns	**3.6 ± 1.8***
	Number of G	0.6 ± 0.2	0.4 ± 0.1ns	0.2 ± 0.1ns

Means ± standard error, *P<0.05; **P<0.01; ***P<0.001 (Mann-Whitney U pairwise comparisons: *wrky22-3* versus Col-0 and *wrky22-4* versus Col-0. NP = non-penetration, C = pathway, , F = penetration difficulties, E1 = phloem salivation, E2 = phloem ingestion, E2s = sustained phloem ingestion (> 10 min), G = xylem ingestion. Single E1s were phloem salivations that were not directly followed or preceded by phloem uptake. Latency to the vascular bundle was calculated as the time from the start of the recording to the first contact with either a phloem or xylem vessel.

Table S4. Aphid feeding behaviour, measured by 8-hour EPG recordings on wild type (Col-0) and *wrky22* inducible overexpression lines (OE.c and OE.e). *WRKY22* overexpression was induced by supplying estradiol solution to all plants 24 hours before the experiment.

Tissue	Variable	Col-0	OE.c	OE.e
Epidermis/mesophyll	Total duration NP (min)	171.6 ± 20.5	178.5 ± 20ns	160.6 ± 16.4ns
	Latency to 1st C (min)	16.3 ± 3.4	12.6 ± 2.6ns	17 ± 4.7ns
	Total duration C (min)	179.9 ± 9.6	164.6 ± 8.2ns	158.1 ± 8.4ns
	Number of C	43.2 ± 4.7	39.5 ± 3.8ns	39.4 ± 3.3ns
	Number of C < 3 min	30.3 ± 4.5	27.5 ± 3.9ns	27.7 ± 3ns
	Number of C < 20 sec	13.1 ± 2.5	11.7 ± 2.5ns	12.1 ± 1.7ns
	Mean duration C (min)	5 ± 0.6	4.7 ± 0.4ns	4.3 ± 0.3ns
	Total duration F (min)	14.2 ± 8.8	8.3 ± 4.8ns	0.7 ± 0.6ns
	Number of F	0.5 ± 0.3	0.6 ± 0.2ns	0.2 ± 0.1ns
Vascular bundle	Latency to 1st E1 after C (min)	139.5 ± 21	94.9 ± 19ns	104.6 ± 13.2ns
	Mean latency E1 after C (min)	13.2 ± 1.2	11.5 ± 0.7ns	13.6 ± 1.4ns
	Total duration E1 (min)	6.6 ± 0.7	8.3 ± 1.2ns	8.5 ± 1.2ns
	Number of E1	8.9 ± 1.3	11.6 ± 1.2ns	9.6 ± 1ns
	Mean duration E1 (min)	0.8 ± 0.1	0.7 ± 0ns	0.9 ± 0.1ns
	Number of single E1	1 ± 0.2	0.8 ± 0.3ns	1 ± 0.3ns
	E1 in E (%)	11.3 ± 2.9	9.3 ± 1.6ns	7.4 ± 1.4ns
	Latency to 1st E2 (min)	170.3 ± 24	**108.8 ± 18.7***	130.9 ± 16.9ns
	Total duration E2 (min)	107.7 ± 23.4	120.3 ± 17.1ns	150.1 ± 17.8ns
	Number of E2	6.8 ± 1.1	9.4 ± 1ns	7.6 ± 1ns
	Mean duration E2 (min)	19.2 ± 5.2	12.5 ± 1.7ns	25.4 ± 4.5ns
	Total duration E2s (min)	91.7 ± 24.3	89.6 ± 15.5ns	131 ± 17.9ns
	Aphids with E2 (%)	100 ± 0	100 ± 0ns	100 ± 0ns
	E2/C ratio	0.7 ± 0.2	0.8 ± 0.1ns	1 ± 0.2ns
	Latency to vascular bundle (min)	155.7 ± 21.2	**107.5 ± 18.8***	118.6 ± 14.7ns
	Total duration G (min)	0 ± 0	0 ± 0ns	2.1 ± 2ns
	Number of G	0 ± 0	0 ± 0ns	0.1 ± 0.1ns

Means ± standard error, *P<0.05; **P<0.01; ***P<0.001 (Mann-Whitney U pairwise comparisons: OE.c versus Col-0 and OE.e versus Col-0. NP = non-penetration, C = pathway, , F = penetration difficulties, E1 = phloem salivation, E2 = phloem ingestion, E2s = sustained phloem ingestion (> 10 min), G = xylem ingestion. Single E1s were phloem salivations that were not directly followed or preceded by phloem uptake. Latency to the vascular bundle was calculated as the time from the start of the recording to the first contact with either a phloem or xylem vessel.
